# The Changes of T-Wave Amplitude and Tp-Te Interval in the Supine and Standing Electrocardiograms of Pediatric Postural Orthostatic Tachycardia Syndrome and Their Predictive Value for the Intervention Effect of Metoprolol

**DOI:** 10.3390/jcm15051798

**Published:** 2026-02-27

**Authors:** Shuo Wang, Ting Zhao, Fang Li, Yuwen Wang, Hong Cai, Liqun Liu, Chuan Wen, Runmei Zou, Cheng Wang

**Affiliations:** Department of Pediatric Cardiovasology, Children’s Medical Center, The Second Xiangya Hospital, Central South University, No. 139 Renmin Middle Road, Changsha 410011, China; wangshuo998@sina.com (S.W.); wangyw@csu.edu.cn (Y.W.);

**Keywords:** postural orthostatic tachycardia syndrome, electrocardiography, T-wave amplitude, Tp-Te interval, metoprolol, children

## Abstract

**Objective:** To investigate the changes in T-wave amplitude and Tp-Te interval on supine and standing electrocardiograms (ECGs) in pediatric postural orthostatic tachycardia syndrome (POTS), and to explore their predictive value for the therapeutic effect of metoprolol. **Methods:** A total of 59 children diagnosed with POTS who presented with syncope or pre-syncopal symptoms were enrolled as the POTS group, and 52 healthy children served as the control group. Supine and standing ECGs were recorded for all subjects, and T-wave amplitude and Tp-Te interval were measured. Children with POTS were followed-up after metoprolol treatment and divided into a therapeutic response group and a non-response group. **Results:** (1) Comparison of supine vs. standing ECGs: In the POTS group, standing posture (compared with supine posture) was associated with increased heart rate (HR), decreased T-wave amplitude in leads II, III, aVF, V4, V5, and V6, shortened Tp-Te interval in leads I, II, III, aVR, aVF, V1, V3, V4, V5, and V6, and elevated Tp-Te/QT ratio in leads aVL and V5 (all *p* < 0.05). (2) Comparison with the control group: The POTS group exhibited a greater HR difference (ΔHR), as well as larger differences in T-wave amplitude (ΔT-wave amplitude) between supine and standing positions in leads II, aVR, aVL, aVF, V3, and V5 (all *p* < 0.05). (3) Follow-up: Compared with the non-response group, the therapeutic response group showed larger ΔT-wave amplitude in leads III, aVF, V2, V3, V4, and V5, larger Tp-Te interval difference (ΔTp-Te interval) in lead V3, and larger Tp-Te/QT ratio difference (ΔTp-Te/QT ratio) in lead V3 (all *p* < 0.05). (4) Receiver operating characteristic curve: ΔT-wave amplitude in leads III, aVF, V2, V3, V4, and V5, ΔTp-Te interval in lead V3, and ΔTp-Te/QT ratio in lead V3 all had predictive value for the therapeutic effect of metoprolol in pediatric POTS (all *p* < 0.05). **Conclusions:** ΔHR and ΔT-wave amplitude in lead V5 between supine and standing positions are independent risk factors for pediatric POTS. A combination of five indicators—ΔT-wave amplitude in leads V2, V3, and V5, ΔTp-Te interval in lead V3, and ΔTp-Te/QT ratio in lead V3 between supine and standing ECGs—exerts a good predictive effect on the therapeutic response of pediatric POTS to metoprolol intervention.

## 1. Introduction

Postural orthostatic tachycardia syndrome (POTS) is characterized by an excessive increase in heart rate (HR) upon standing, accompanied by symptoms such as dizziness, blurred vision, palpitations, fatigue, abdominal pain, sleep disorders, and migraines following rapid postural changes (body position quickly changing to a standing position from supine or sitting or squatting) or prolonged standing. These symptoms are alleviated after recumbency [1,2]. The prevalence of POTS is 0.2%, predominantly affecting individuals aged 15–25 years, with 75% of cases occurring in females [3]. Currently, POTS is mostly classified as a “functional cardiovascular disease”. However, recurrent symptoms can adversely affect children’s physical and mental health and quality of life; in severe cases, syncope-related physical injuries may occur [4,5]. The pathogenesis of POTS includes orthostatic central hypovolemia, autonomic dysfunction, hyperadrenergic state, vasomotor dysfunction, abnormal immune function, or a combination of them [6,7]. Beta-blockers (e.g., metoprolol) are commonly used in the treatment of pediatric POTS, as they can reduce sympathetic nerve activity and/or lower plasma catecholamine levels [8,9]. However, clinical practice has shown that metoprolol only alleviates symptoms in a subset of pediatric POTS patients, with an effective rate of approximately 50% [10]. Ladage et al. [11] proposed that metoprolol may impair motor tolerance in children. Therefore, predicting the efficacy of metoprolol in pediatric POTS prior to medication administration has become a clinical need.

Previous studies have identified several biomarkers with predictive value for the therapeutic effect of metoprolol in pediatric POTS [12], including orthostatic plasma norepinephrine level [13], plasma copeptin [14], C-type natriuretic peptide [15], 24 h heart rate variability [16], Poincare plot [17], HR changes during the head-up tilt test (HUTT) [18], corrected QT interval dispersion [19], and corrected *p*-wave maximum, corrected minimum QT interval, and Tp-Te interval dispersion [20]. However, the acquisition of these indicators is complex and associated with high testing costs. Thus, there is a need to identify simple, low-cost, and non-invasive biomarkers for predicting the efficacy of metoprolol intervention in pediatric POTS.

Cardiac activity is regulated by the sympathetic and vagus nerves, and electrocardiogram (ECG) waveforms can reflect the effects of the interaction between these two nervous systems on cardiac function. Changes in supine and standing ECG waveforms are associated with alterations in autonomic nervous function T-wave amplitude and Tp-Te interval on ECG, reflecting ventricular repolarization changes, and are important indicators for evaluating cardiac autonomic nervous function [21]. To date, no studies have reported whether changes in supine and standing ECG waveforms have predictive value for the efficacy of metoprolol in pediatric POTS. This study aims to explore the predictive value of these ventricular repolarization indicators for metoprolol’s therapeutic effect in pediatric POTS by analyzing changes in T-wave amplitude, Tp-Te interval, and Tp-Te/QT ratio between supine and standing ECGs.

## 2. Objects and Methods

### 2.1. Research Objects

**Figure 1:**
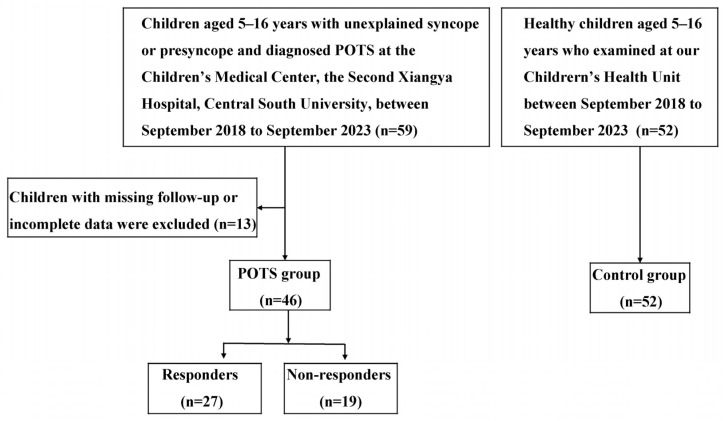
Flow Chart.

Children with syncopal or pre-syncopal symptoms who attended The Second Xiangya Hospital, Central South University, from September 2018 to September 2023 were enrolled. Through detailed medical history inquiry, physical examination, hematological tests (complete blood count, liver and kidney function, electrolytes, myocardial enzymes, blood glucose, blood lipids, thyroid function, etc.), and imaging examinations (12-lead ECG, Holter ECG, 24 h ambulatory blood pressure monitoring, echocardiography, cardiac X-ray, electroencephalography, head CT or MRI, etc.), organic diseases of the heart, brain, lungs, and blood vessels, immune diseases, and psychological disorders were excluded. A total of 59 children aged 5–16 years diagnosed with POTS via HUTT were included in the POTS group (40 males, age 11.82 ± 2.25 years). During the same period, 52 healthy children matched by age and sex were selected as the control group (26 males, age 11.55 ± 1.68 years). Thirteen pediatric POTS patients were excluded due to lost follow-up or incomplete data. Based on the therapeutic effect of metoprolol, the remaining 46 POTS patients were divided into a treatment response group and a non-response group (Figure 1). This study was approved by the Medical Ethics Committee of the Second Xiangya Hospital, Central South University (Ethical Audit No. Study 249(2022)), in compliance with the principles of the Declaration of Helsinki. Written informed consent was obtained from all participants and/or their guardians.

### 2.2. Research Methods

#### 2.2.1. Basic Head-Up Tilt Test (BHUT) [2]

BHUT was performed between 8:00 a.m. and 11:00 a.m. in a quiet, dimly lit room with an ambient temperature of 22–24 °C. Vasoactive medications were discontinued for at least five half-lives prior to the test, and subjects fasted (no food or water intake) for 4 h before the test. After emptying their bladders, subjects lay supine on a tilt table for 10 min. The tilt devices used were the Head-up Tilt Test System (ST-711, Beijing Juchi Medical Technology Co., Ltd., Beijing, China) and the Head-up Tilt Test Monitoring System (SHUT-100, Jiangsu Standard Medical Technology Co., Ltd., Wuxi, China). Blood pressure (BP), HR, and ECG were recorded. The tilt table was adjusted from the supine position to a 60° head-up, feet-down position within 15 s. BP, HR, ECG, and clinical manifestations of the subjects were continuously monitored until a positive response occurred or the 45 min test was completed.

#### 2.2.2. POTS Diagnostic Criteria

The diagnostic criteria for POTS were as follows [2]: (1) disease duration > 1 month, often associated with most of the aforementioned inducing factors, such as rapid postural changes or prolonged standing, etc.; (2) presence of orthostatic intolerance symptoms, including dizziness, headache, fatigue, blurred vision, chest tightness, palpitations, hand tremors, exercise intolerance, and even syncope, particularly in the standing position; (3) positive HUTT result: during the first 10 min of HUTT, a positive POTS response is defined as an increase in HR ≥ 40 bpm, or a maximum standing HR ≥ 130 bpm (for children aged 5–12 years) or ≥125 bpm (for adolescents aged 12–18 years). All positive responses must exclude a significant decrease in BP (systolic BP decrease > 20 mmHg and/or diastolic BP decrease > 10 mmHg); (4) exclusion of other diseases that may cause similar symptoms.

#### 2.2.3. Electrocardiogram Recording [21,22]

Cardioactive medications and drugs affecting autonomic nervous function were discontinued for five half-lives prior to ECG recording. Subjects lay quietly on the examination table, and supine synchronous 12-lead ECG was recorded using an MAC800 ECG machine (GE Medical System, Shanghai, China). Subjects then stood upright with unchanged electrode positions, and standing synchronous 12-lead ECG was recorded once the waveform stabilized. No filtering devices were used during sampling. The gain was set to 1 mV = 10 mm, and the paper speed was 25 mm/s. T-wave amplitude and Tp-Te interval were measured in all 12 leads.

Measurement methods: baseline ECG measurement during POTS diagnosis. (1) T-wave amplitude measurement: the Q-wave onset was used as the reference horizontal line [23]. For positive T-waves, amplitude was defined as the vertical distance from the upper edge of the reference line to the waveform peak; for negative T-waves, it was the vertical distance from the lower edge of the reference line to the waveform trough; for bidirectional T-waves, it was the algebraic sum of positive and negative amplitudes. (2) Tp-Te interval measurement: the time interval from the T-wave peak to the T-wave end. The T-wave end was determined by one of the following methods: ① the intersection of the T-wave and the isoelectric line; ② the tangent line between the T-wave and U-wave; ③ the final intersection of the bidirectional T-wave with the isoelectric line. All indicators were measured in a double-blind manner over three cardiac cycles, and the average value was calculated. Sinus rhythm and clear waveforms were required for measurement. The difference between supine and standing parameters in the same lead was calculated as the absolute value of the supine parameter minus the standing parameter. HR difference, T-wave amplitude difference, Tp-Te interval difference, and Tp-Te/QT ratio difference were, respectively, represented as ΔHR, ΔT-wave amplitude, ΔTp-Te interval, and ΔTp-Te/QT ratio.

#### 2.2.4. Symptom Score (SS)

SS was developed based on the frequency of dizziness, syncope, headache, chest tightness, palpitations, sweating, nausea, hand tremors, blurred vision, and inattention [24]. Scoring criteria: 0 points = asymptomatic; 1 point = symptoms occur once a month; 2 points = symptoms occur 2–4 times a month; 3 points = symptoms occur 2–7 times a week; 4 points = symptoms occur more than once a day. The total SS was the sum of scores for individual symptoms. SS assessed at the time of POTS diagnosis was defined as pre-treatment SS, and SS assessed during the first follow-up visit (median 55.77 days after treatment initiation) was defined as post-treatment SS.

#### 2.2.5. Treatment and Follow-Up

Pediatric POTS patients were administered metoprolol tablets [0.5–1 mg/(kg·d), orally, twice daily] [18,24] and followed-up for a median of 55.77 (interquartile range: 20.56, 100) days after treatment (metoprolol tablets, 25 mg/tablet, AstraZeneca Pharmaceutical Co., Ltd., Wuxi, China, National Drug Approval No. H32025391). Therapeutic response was uniformly evaluated at the first follow-up visit (clinically planned as 4–8 weeks post-treatment to ensure sufficient drug exposure). Based on therapeutic efficacy, patients were divided into two groups: (1) treatment response group: post-treatment SS decreased by ≥2 points compared with pre-treatment, or improved HUTT results on re-examination; (2) treatment non-response group: post-treatment SS decreased by <2 points compared with pre-treatment, or no improvement in HUTT results on re-examination.

#### 2.2.6. Statistical Analysis

Data were analyzed using EmpowerStats 4.0 and SPSS 26.0 software. The Shapiro–Wilk test was used to assess the normality of continuous data. Normally distributed data were expressed as mean ± standard deviation ($\bar{x}$ ± s), while non-normally distributed data were expressed as median [interquartile range, *M (P25, P75)*]. Categorical data were expressed as case numbers (%). Comparisons between groups were performed using *t*-test, nonparametric test, *χ*^2^ test, Fisher’s exact test, or the Mann–Whitney U test. Multivariate analysis was conducted using logistic regression analysis. Receiver operating characteristic (ROC) curves and the area under the curve (AUC) were used to evaluate the diagnostic efficacy and predictive value of the observed indicators. A *p* value < 0.05 was considered statistically significant.

## 3. Results

### 3.1. Comparison of Supine and Standing ECG Parameters in the POTS Group

Compared with the supine position, the standing HR was significantly increased in the POTS group (108.68 ± 15.73 bpm vs. 79.10 ± 14.96 bpm, *p* < 0.05). Additionally, the T-wave amplitude was decreased in leads II, III, aVF, V4, V5, and V6 (all *p* < 0.05); the Tp-Te interval was shortened in leads I, II, III, aVR, aVF, V1, V3, V4, V5, and V6 (all *p* < 0.05); and the Tp-Te/QT ratio was increased in leads aVL and V5 (all *p* < 0.05) (Table 1).

### 3.2. Comparison of ECG Parameters Between the POTS Group and the Control Group

**Supine ECG**: There was no significant difference in HR between the POTS group and the control group (79.10 ± 14.96 bpm vs. 76.69 ± 12.62 bpm, *p* > 0.05). However, the Tp-Te interval was prolonged in leads I, V1, V4, and V5, and the Tp-Te/QT ratio was increased in lead V4 in the POTS group compared with the control group (all *p* < 0.05). No statistically significant differences in T-wave amplitude of any lead were observed between the two groups (all *p* > 0.05) (Table 2).

**Standing ECG**: The HR in the POTS group was significantly higher than that in the control group (108.68 ± 15.73 bpm vs. 100.36 ± 11.75 bpm, *p* < 0.05). In addition, the T-wave amplitude in lead III was decreased, and the Tp-Te interval in lead V5 was prolonged in the POTS group compared with the control group (all *p* < 0.05). No statistically significant differences in Tp-Te/QT ratio of any lead were found between the two groups (all *p* > 0.05) (Table 2).

**Supine and standing ECGs**: Compared with the control group, the POTS group exhibited a significantly larger ΔHR between supine and standing positions (29.58 ± 14.64 bpm vs. 20.74 ± 11.57 bpm, *p* < 0.05) and larger ΔT-wave amplitude in leads aVR, aVL, aVF, V3, and V5 (all *p* < 0.05). No statistically significant differences were observed in the ΔTp-Te interval or ΔTp-Te/QT ratio of any lead between the two groups (all *p* > 0.05) (Table 2).

### 3.3. Logistic Regression Analysis

Multivariate logistic regression analysis was performed on parameters with significant differences in supine-standing ECG between the POTS group and the control group, including ΔHR and ΔT-wave amplitude in leads II, aVR, aVL, aVF, V3, and V5. After adjusting for demographic factors, ΔHR and ΔT-wave amplitude in lead V5 were identified as independent risk factors for POTS. Specifically, for every 1 bpm increase in ΔHR, the risk of POTS increased by 5%; for every 1 mV increase in ΔT-wave amplitude in lead V5, the risk of POTS increased by 33.37% (Table 3).

### 3.4. Comparison of ECG Parameters Between the Metoprolol-Responsive and Non-Responsive POTS Groups

Among the 59 children with POTS, 46 completed follow-up. General information of the responsive group (27 cases, 58.7%) and non-responsive group (19 cases, 41.3%) is shown in Table 4. There was no significant difference in ΔHR between the two groups (33.88 ± 17.73 bpm vs. 27.35 ± 11.74 bpm, *p* > 0.05). However, the responsive group had larger ΔT-wave amplitude in leads III, aVF, V2, V3, V4, and V5, larger ΔTp-Te interval in lead V3, and larger ΔTp-Te/QT ratio in lead V3 compared with the non-responsive group (all *p* < 0.05) (Table 4).

### 3.5. Receiver Operating Characteristic (ROC) Curve

**Figure 2:**
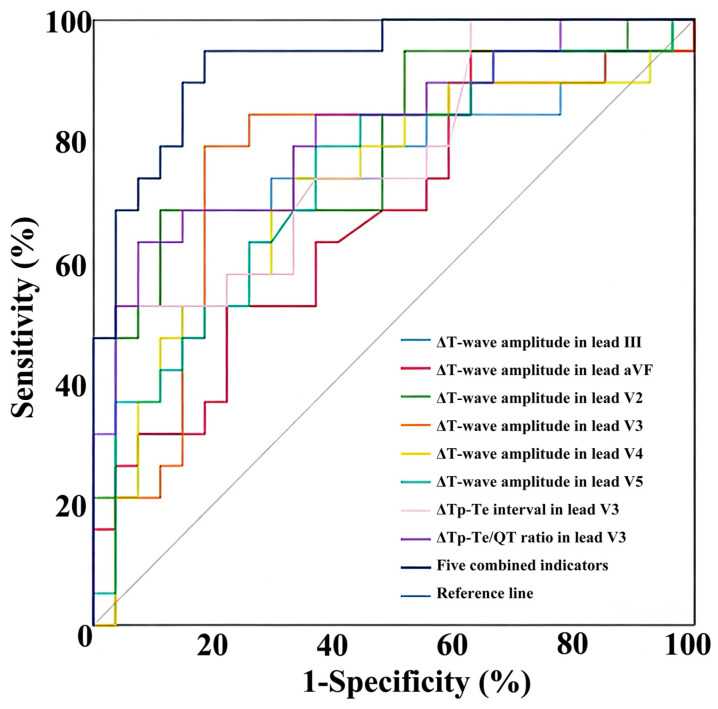
ROC curves of ventricular repolarization parameters in supine and standing ECGs for the prognosis of POTS to metoprolol.

The area under the curve (AUC) values of ΔT-wave amplitude in leads III, aVF, V2, V3, V4, and V5, ΔTp-Te interval in lead V3, and ΔTp-Te/QT ratio in lead V3 for predicting the therapeutic effect of metoprolol in POTS were 0.70, 0.67, 0.79, 0.75, 0.72, 0.74, 0.74, and 0.82, respectively. To improve predictive performance, a combination of five indicators was constructed: ΔT-wave amplitude in leads V2, V3, and V5, ΔTp-Te interval in lead V3, and ΔTp-Te/QT ratio in lead V3 between supine and standing positions. This combined indicator significantly enhanced the predictive ability for metoprolol’s therapeutic effect, with an AUC of 0.93, sensitivity of 94.70%, and specificity of 81.50% (Table 5, Figure 2).

To assess potential overfitting and validate model robustness, bootstrapping internal validation (1000 resamples) was performed. The corrected AUC was 0.91 (95% CI: 0.86–0.96), with a minimal value of 0.02. The corrected sensitivity and specificity were 92.30% and 79.80%, respectively, which are closely consistent with the original values, indicating the model’s stability and low risk of overfitting.

## 4. Discussion

Autonomic nervous function is closely associated with the cardiovascular system [25]. It primarily affects myocardial depolarization and repolarization processes by secreting neurotransmitters and altering ion distribution on the myocardial cell membrane surface [26,27], thereby regulating ECG waveform changes.

The T-wave is a key indicator reflecting ventricular repolarization on ECG, corresponding to phase 3 of the myocardial action potential. Ventricular repolarization is regulated by the autonomic nervous system: cardiac sympathetic nerves are mainly distributed under the epicardium, and their release of norepinephrine binds to β-adrenergic receptors, phosphorylating ion channel proteins, altering channel opening probability, accelerating potassium ion outflow during repolarization, shortening the repolarization process and effective refractory period, which may manifest as low, inverted, or bimodal T-waves. In contrast, the vagus nerve is mainly distributed under the ventricular endocardium, and its activation can result in peaked T-waves [28]. Pediatric POTS patients exhibit elevated norepinephrine levels, which are position-dependent: plasma norepinephrine concentration increases rapidly upon standing, leading to sustained elevation of sympathetic nerve activity [29]. Kanjwal et al. [30] reported that symptomatic pediatric POTS occurs when plasma norepinephrine levels reach ≥600 pg/mL. Postural changes are a common trigger for reflexive regulation of cardiac autonomic nervous function [31].

In the present study, the POTS group had a higher standing HR and lower T-wave amplitude in lead III compared with the control group. This may be attributed to increased β-receptor reactivity, enhanced sympathetic excitability, increased myocardial cell membrane channel opening, and accelerated repolarization potassium outflow during the supine-to-standing transition, leading to rapid phase 3 repolarization of the action potential and consequent flattened or inverted T-waves. Li et al. [28] demonstrated that HUTT-positive patients have larger supine–standing ΔT-wave amplitude than HUTT-negative patients, particularly in leads II, III, aVF, and V5, confirming that postural changes in T-wave amplitude can serve as a marker of autonomic nervous dysfunction. Wang et al. [22] reported that a combined indicator of supine–standing ECG ΔHR ≥15 bpm and ΔT-wave amplitude ≥0.10 mV in leads V5 and V6 has predictive value for pediatric POTS diagnosis. However, our findings diverge from the conclusion that supine and standing ECG parameters cannot predict metoprolol’s therapeutic efficacy in pediatric POTS. This discrepancy arises because the prior study employed a combined intervention strategy, incorporating both nonpharmacologic and pharmacologic treatments, and did not assess the ΔTp-Te interval or the ΔTp-Te/QT ratio as outcome measures.

Consistent with previous studies, our results showed that pediatric POTS patients have increased standing HR, decreased T-wave amplitude in multiple leads (predominantly II, III, aVF, V4, V5, and V6), larger ΔHR, and more significant decreases in ΔT-wave amplitude (leads II, aVF, V3, and V5) compared with the control group after postural change. These findings suggest poor stability of autonomic nervous function in pediatric POTS, with more pronounced autonomic imbalance during postural transitions. Multivariate logistic regression analysis further confirmed that ΔHR and ΔT-wave amplitude in lead V5 are independent risk factors for POTS after adjusting for demographic factors. Additionally, metoprolol-responsive POTS patients had greater decreases in T-wave amplitude in leads III, aVF, V2, V3, V4, and V5 than non-responsive patients, indicating that responders may have higher sympathetic activity and norepinephrine levels after postural change, supporting the efficacy of individualized metoprolol therapy in this subgroup. Significant differences exist in electrical activity among myocardial cells in different ventricular regions and layers, primarily manifested as marked transmural repolarization heterogeneity of the three myocardial layers during repolarization [32]. The Tp-Te interval refers to the time from the T-wave peak to the T-wave end on ECG; its prolongation indicates increased repolarization dispersion and has predictive value for common ion channel diseases associated with malignant ventricular arrhythmias, such as long QT syndrome, short QT syndrome, and Brugada syndrome [33,34,35]. The Tp-Te/QT ratio reflects the proportion of the Tp-Te interval in the repolarization process and can eliminate the influence of HR and individual QT interval variability [36].

Yagishita et al. [37] reported that sympathetic nerve stimulation of one or both stellate ganglia in pigs significantly prolongs the Tp-Te interval. Tanabe et al. [38] found that high catecholamine levels significantly increase Tp-Te interval dispersion in long QT syndrome patients, indicating a close association between Tp-Te interval changes and high catecholamine levels/sympathetic activity. Patients with autonomic nervous dysfunction exhibit increased ventricular repolarization electrical heterogeneity and abnormal Tp-Te intervals. Amoozgar et al. [39] reported that HUTT-positive children with syncope have a longer Tp-Te interval in lead V1 than HUTT-negative children.

In the current study, the POTS group had prolonged Tp-Te intervals in leads I, V1, V4, and V5, and an increased Tp-Te/QT ratio in lead V4 compared with the control group in both supine and standing ECGs, suggesting increased ventricular repolarization heterogeneity in pediatric POTS. However, no significant differences in ΔTp-Te interval or ΔTp-Te/QT ratio were observed between the two groups, which may be attributed to insufficient standing time during ECG recording (resulting in inadequate autonomic nervous system stimulation) or the transient nature of autonomic dysfunction-induced ECG changes. Xu et al. [20] followed-up pediatric POTS patients treated with metoprolol and found that responders had a longer maximum Tp-Te interval and greater Tp-Te interval dispersion than non-responders, indicating the predictive value of the Tp-Te interval for POTS prognosis.

Consistent with this, our study showed that metoprolol-responsive POTS patients had larger ΔTp-Te interval and ΔTp-Te/QT ratio in lead V3 than non-responders, suggesting higher pre-treatment sympathetic activity in responders. ROC curve analysis further confirmed that the combined indicator of ΔT-wave amplitude in leads V2, V3, and V5, ΔTp-Te interval in lead V3, and ΔTp-Te/QT ratio in lead V3 has excellent predictive value for metoprolol’s therapeutic effect in pediatric POTS, indicating that the combined T-wave amplitude and Tp-Te interval parameters can serve as reliable predictors of metoprolol efficacy in POTS. However, ventricular repolarization indices derived from ECGs might compromise the consistency of therapeutic response assessments in pediatric POTS treated with metoprolol, particularly when nonstandardized follow-up intervals are employed. These findings warrant validation in prospective studies conducted under standardized, harmonized protocols.

## 5. Limitations

This study has several limitations: (1) the sample size was limited due to the single-center design, which might lead to potential bias; (2) the standing time during ECG recording was mostly approximately 3 min, while the median time to reach maximum HR in the active standing test is 5 min [40]. Therefore, extending the standing time in future studies may more sufficiently stimulate autonomic nervous system changes; (3) for patients with POTS receiving treatment, there is currently no unified follow-up protocol, resulting in considerable variability in follow-up duration (median: 55.77 days; interquartile range: 20.56 to 100 days). This variability may compromise the accuracy of treatment efficacy assessment, and constitutes one of the inherent limitations of the retrospective study design. (4) This retrospective study had a long follow-up time span, and treatment efficacy might be influenced by factors such as treatment compliance and duration, potentially introducing bias.

## 6. Conclusions

ΔHR between supine and standing ECGs and ΔT-wave amplitude in lead V5 are independent risk factors for pediatric POTS. The combined indicator of five parameters—ΔT-wave amplitude in leads V2, V3, and V5, ΔTp-Te interval in lead V3, and ΔTp-Te/QT ratio in lead V3 between supine and standing ECGs—has excellent predictive value for the therapeutic effect of metoprolol in pediatric POTS. These findings provide valuable guidance for formulating individualized treatment plans for pediatric POTS.

## Figures and Tables

**Table 1:** Comparison of T-wave amplitude, Tp-Te interval, and Tp-Te/QT ratio between supine and standing ECGs in the POTS group [($\bar{x}$ ± s) or *M (P25, P75)*].

Lead	T-Wave Amplitude (mV)				Tp-Te Interval (ms)				Tp-Te/QT Ratio			
	Supine ECG (n = 46)	Standing ECG (n = 46)	*t*/*Z*	*p* Value	Supine ECG (n = 46)	Standing ECG (n = 46)	*t*/*Z*	*p* Value	Supine ECG (n = 46)	Standing ECG (n = 46)	*t*/*Z*	*p* Value
I	0.35 (0.28, 0.44)	0.32 (0.25, 0.38)	1.68	0.060	74.95 ± 9.33	67.15 ± 11.52	4.04	0.000	0.21 ± 0.03	0.21 ± 0.03	0.08	0.786
II	0.39 (0.32, 0.53)	0.22 (0.17, 0.31)	6.17	0.000	77.29 ± 10.49	68.59 ± 15.24	3.61	0.000	0.21 (0.19, 0.23)	0.20 (0.18, 0.23)	0.21	0.204
III	0.12 (−0.09, 0.19)	−0.16 (−0.22, −0.10)	7.29	0.000	66.38 ± 13.92	57.25 ± 13.30	3.64	0.000	0.18 (0.16, 0.20)	0.18 (0.16, 0.19)	1.07	0.145
aVR	−0.38 (−0.49, −0.30)	−0.28 (−0.33, −0.21)	−4.73	0.000	75.13 ± 10.75	64.99 ± 12.73	4.68	0.000	0.21 ± 0.03	0.20 ± 0.04	0.95	0.264
aVL	0.16 (0.11, 0.23)	0.22 (0.18, 0.29)	−4.59	0.000	63.05 ± 11.19	62.00 ± 9.77	0.54	0.889	0.18 ± 0.03	0.19 ± 0.03	−2.46	0.011
aVF	0.27 (0.18, 0.36)	0.11 (−0.11, 0.18)	6.25	0.000	69.19 ± 11.60	59.97 ± 8.53	4.92	0.000	0.19 (0.18, 0.21)	0.19 (0.17, 0.19)	1.03	0.135
V1	−0.17 (−0.27, −0.08)	−0.16 (−0.24, −0.10)	0.17	0.970	65.00 (55.00, 74.00)	57.00 (49.00, 62.00)	2.88	0.002	0.18 (0.16, 0.20)	0.18 (0.15, 0.19)	0.12	0.615
V2	0.23 (−0.19, 0.43)	0.15 (−0.18, 0.36)	0.45	0.581	78.46 (56.00, 96.00)	62.00 (53.00, 91.00)	1.70	0.078	0.22 (0.16, 0.25)	0.21 (0.16, 0.26)	0.02	0.769
V3	0.32 (0.06, 0.61)	0.23 (−0.13, 0.42)	1.66	0.073	88.00 (71.00, 96.00)	72.00 (54.00, 94.00)	2.10	0.022	0.23 ± 0.05	0.23 ± 0.07	−0.76	0.718
V4	0.45 (0.35, 0.77)	0.30 (0.16, 0.43)	4.59	0.000	84.61 ± 12.77	76.25 ± 21.94	2.53	0.013	0.23 ± 0.04	0.24 ± 0.06	−1.21	0.202
V5	0.61 (0.36, 0.73)	0.31 (0.19, 0.38)	6.90	0.000	79.00 (71.00, 85.00)	72.00 (60.00, 82.00)	1.69	0.018	0.21 ± 0.03	0.23 ± 0.05	−2.00	0.048
V6	0.43 (0.30, 0.56)	0.25 (0.18, 0.33)	6.46	0.000	72.56 ± 12.22	65.68 ± 13.90	2.86	0.001	0.20 (0.18, 0.22)	0.20 (0.18, 0.22)	−0.41	0.832

**Table 2:** Comparison of ECG T-wave amplitude, Tp-Te interval, and Tp-Te/QT ratio between the POTS group and the control group [($\bar{x}$ ± s) or *M (P25, P75)*].

Lead	T-Wave Amplitude (mV)				Tp-Te Interval (ms)				Tp-Te/QT Ratio			
	Control Group (n = 52)	POTS Group (n = 46)	*t*/*Z*	*p* Value	Control Group (n = 52)	POTS Group (n = 46)	*t*/*Z*	*p* Value	Control Group (n = 52)	POTS Group (n = 46)	*t*/*Z*	*p* Value
Supine ECG												
I	0.35 (0.26, 0.44)	0.35 (0.28, 0.44)	−0.68	0.497	71.23 ± 11.49	74.95 ± 9.33	−2.22	0.026	0.20 ± 0.03	0.21 ± 0.03	−1.05	0.297
II	0.42 ± 0.16	0.43 ± 0.17	−0.23	0.820	73.79 ± 8.93	77.29 ± 10.49	−1.89	0.059	0.21 ± 0.02	0.21 ± 0.03	−0.89	0.376
III	0.12 (−0.04, 0.21)	0.14 (−0.09, 0.21)	−0.02	0.988	61.72 ± 14.87	66.38 ± 13.92	−1.51	0.131	0.18 ± 0.04	0.19 ± 0.04	−1.22	0.227
aVR	−0.36 (−0.48, −0.28)	−0.38 (−0.49, −0.30)	−0.53	0.599	72.31 ± 11.27	75.13 ± 10.75	−1.34	0.181	0.20 ± 0.03	0.21 ± 0.03	−0.58	0.563
aVL	0.14 (0.11, 0.22)	0.16 (0.11, 0.22)	−0.53	0.597	60.47 ± 12.27	63.05 ± 11.19	−1.17	0.241	0.18 ± 0.04	0.18 ± 0.03	−0.78	0.437
aVF	0.27 ± 0.12	0.26 ± 0.16	−0.29	0.774	66.88 ± 9.85	69.19 ± 11.60	−1.20	0.229	0.19 ± 0.03	0.19 ± 0.03	−0.77	0.444
V1	−0.18 (−0.27, −0.10)	−0.17 (−0.27, −0.08)	−0.53	0.595	60.35 ± 14.93	65.31 ± 15.30	−2.02	0.044	0.17 ± 0.04	0.18 ± 0.04	−0.99	0.325
V2	0.22 (−0.13, 0.30)	0.23 (−0.19, 0.43)	−0.03	0.976	67.50 (55.00, 85.50)	78.46 (56.00, 95.50)	−1.30	0.192	0.21 ± 0.06	0.21 ± 0.06	−0.81	0.419
V3	0.32 (0.23, 0.45)	0.32 (0.06, 0.61)	−0.16	0.876	81.46 ± 18.13	83.02 ± 18.84	−0.56	0.574	0.23 ± 0.05	0.23 ± 0.05	0.20	0.842
V4	0.44 (0.35, 0.54)	0.45 (0.35, 0.77)	−0.84	0.401	77.04 ± 11.36	84.61 ± 12.77	−3.42	0.000	0.22 ± 0.03	0.23 ± 0.04	−2.20	0.030
V5	0.48 (0.36, 0.60)	0.61 (0.36, 0.73)	−1.46	0.144	72.85 ± 8.73	77.49 ± 10.47	−2.74	0.006	0.21 ± 0.02	0.21 ± 0.03	−1.30	0.198
V6	0.41 ± 0.16	0.43 ± 0.17	−0.84	0.400	68.92 ± 9.12	72.56 ± 12.22	−1.54	0.123	0.20 ± 0.02	0.20 ± 0.03	−0.50	0.617
Standing ECG												
I	0.30 (0.22, 0.37)	0.32 (0.25, 0.37)	−1.06	0.289	65.63 ± 10.56	67.15 ± 11.52	−1.33	0.184	0.20 ± 0.03	0.21 ± 0.03	−1.32	0.188
II	0.27 (0.20, 0.35)	0.22 (0.17, 0.31)	−1. 24	0.216	66.46 ± 12.09	68.59 ± 15.24	−0.36	0.707	0.20 ± 0.04	0.21 ± 0.05	−0.27	0.788
III	−0.11 (−0.16, 0.08)	−0.16 (−0.22, −0.10)	−2.88	0.004	62.00 (52.00, 69.00)	57.00 (49.00, 63.00)	−2.02	0.044	0.19 (0.15, 0.22)	0.18 (0.16, 019)	−1.86	0.064
aVR	−0.30 (−0.35, −0.23)	−0.28 (−0.33, −0.21)	−0.48	0.634	67.62 ± 11.35	64.99 ± 12.73	−1.20	0.231	0.21 ± 0.04	0.20 ± 0.04	−0.99	0.322
aVL	0.20 (0.15, 0.26)	0.21 (0.18, 0.28)	−1.87	0.062	60.00 ± 10.98	62.00 ± 9.77	−1.16	0.248	0.19 ± 0.03	0.19 ± 0.03	−1.23	0.218
aVF	0.16 (0.09, 0.23)	0.11 (−0.11, 0.18)	−1.81	0.070	62.38 ± 14.49	59.97 ± 8.53	−1.42	0.156	0.19 (0.16, 0.23)	0.19 (0.17, 0.19)	−1.51	0.131
V1	−0.16 (−0.22, −0.09)	−0.16 (−0.24, −0.10)	−0.42	0.673	53.00 (46.00, 65.00)	57.00 (49.00, 62.00)	−0.08	0.933	0.17 (0.15, 0.20)	0.18 (0.15, 0.19)	−0.17	0.862
V2	0.19 (−0.10, 0.32)	0.15 (−0.18, 0.36)	−0.69	0.489	54.00 (47.00, 73.00)	62.00 (53.00, 91.00)	−1.48	0.140	0.19 (0.15, 0.23)	0.21 (0.16, 0.26)	−1.32	0.186
V3	0.26 (0.18, 0.37)	0.23 (−0.13, 0.42)	−0.57	0.566	69.00 (52.00, 99.00)	72.00 (54.00, 94.00)	−0.15	0.880	0.22 (0.17, 0.29)	0.24 (0.18, 0.29)	−0.33	0.741
V4	0.29 (0.19, 0.41)	0.30 (0.16, 0.43)	−0.32	0.752	76.71 ± 21.07	76.25 ± 21.94	−0.02	0.981	0.24 ± 0.06	0.24 ± 0.06	−0.44	0.658
V5	0.30 (0.21, 0.40)	0.31 (0.19, 0.38)	−0.04	0.965	66.65 ± 14.08	73.10 ± 16.96	−2.05	0.041	0.20 (0.18, 0.24)	0.22 (0.19, 0.26)	−1.93	0.053
V6	0.27 (0.18, 0.36)	0.25 (0.18, 0.33)	−0.30	0.765	61.00 ± 10.40	65.68 ± 13.90	−1.90	0.058	0.19 (0.17, 0.22)	0.20 (0.18, 0.22)	−1.36	0.173
Supine ECG and standing ECG												
I	0.05 (0.03, 0.10)	0.05 (0.02, 0.09)	−0.29	0.770	9.00 (3.75, 15.00)	10.00 (5.00, 16.00)	−0.48	0.634	0.02 (0.01, 0.04)	0.02 (0.01, 0.03)	−0.04	0.967
II	0.12 (0.07, 0.20)	0.18 (0.11, 0.27)	−2.29	0.022	11.00 (5.00, 19.00)	11.00 (4.00, 20.50)	−0.32	0.750	0.02 (0.01, 0.04)	0.03 (0.01, 0.04)	−1.15	0.252
III	0.14 (0.05, 0.25)	0.23 (0.09, 0.32)	−1.96	0.050	11.00 (4.00, 18.00)	14.00 (9.00, 21.64)	−0.98	0.328	0.03 (0.01, 0.07)	0.03 (0.02, 0.05)	0.43	0.667
aVR	0.07 (0.03, 0.13)	0.11 (0.05, 0.15)	−2.13	0.033	7.00 (4.00, 16.50)	10.00 (5.00, 22.50)	−1.78	0.075	0.03 (0.01, 0.03)	0.03 (0.02, 0.04)	−1.12	0.264
aVL	0.05 (0.02, 0.09)	0.07 (0.04, 0.12)	−2.19	0.029	7.00 (3.25, 11.00)	8.00 (5.00, 16.00)	−1.49	0.137	0.02 (0.01, 0.04)	0.02 (0.01, 0.04)	−1.11	0.266
aVF	0.12 (0.05, 0.21)	0.21 (0.10, 0.27)	−2.59	0.010	11.00 (4.00, 24.00)	11.00 (5.50, 20.00)	−0.04	0.967	0.03 (0.01, 0.06)	0.03 (0.01, 0.05)	−1.17	0.241
V1	0.05 (0.03, 0.12)	0.05 (0.02, 0.10)	−0.69	0.491	8.00 (3.00, 11.25)	12.00 (4.00, 19.50)	−1.40	0.161	0.02 (0.01, 0.04)	0.02 (0.01, 0.04)	−0.30	0.762
V2	0.06 (0.04, 0.13)	0.11 (0.04, 0.24)	−1.20	0.231	14.00 (6.00, 26.75)	14.00 (7.00, 28.65)	−0.13	0.894	0.03 (0.02, 0.06)	0.04 (0.01, 0.06)	−0.36	0.723
V3	0.09 (0.04, 0.16)	0.13 (0.06, 0.29)	−2.42	0.016	17.00 (8.50, 29.00)	13.00 (4.65, 28.50)	−0.90	0.367	0.05 (0.02, 0.08)	0.04 (0.02, 0.06)	−1.06	0.287
V4	0.14 (0.11, 0.27)	0.19 (0.11, 0.43)	−1.67	0.096	13.50 (9.00, 20.25)	14.00 (7.00, 26.00)	−0.13	0.894	0.05 (0.02, 0.07)	0.05 (0.02, 0.08)	−0.36	0.723
V5	0.16 (0.10, 0.25)	0.23 (0.15, 0.35)	−2.59	0.010	12.00 (7.00, 20.00)	13.00 (6.00, 20.00)	−0.51	0.608	0.03 (0.01, 0.04)	0.03 (0.01, 0.06)	−0.74	0.459
V6	0.12 (0.06, 0.18)	0.11 (0.06, 0.23)	−0.57	0.579	9.00 (5.25, 15.75)	12.00 (4.50, 21.50)	−1.36	0.173	0.02 (0.01, 0.03)	0.02 (0.01, 0.04)	−1.13	0.258

**Table 3:** Logistic regression analysis for predicting the risk of POTS.

Variable	OR (95% CI)	*p* Value
ΔHR (bpm)	1.05 (1.01, 1.09)	0.007
ΔT-wave amplitude in lead II (mV)	5.96 (0.10, 367.40)	0.396
ΔT-wave amplitude in lead aVR (mV)	22.08 (0.05, 9735.33)	0.319
ΔT-wave amplitude in lead aVL (mV)	142.14 (0.06, 330.75)	0.210
ΔT-wave amplitude in lead aVF (mV)	19.19 (0.48, 761.93)	0.116
ΔT-wave amplitude in lead V3 (mV)	5.70 (0.21, 153.51)	0.300
ΔT-wave amplitude in lead V5 (mV)	34.37 (1.11, 271.21)	0.043

Result variable: POTS. Exposure variable: ΔHR, ΔT-wave amplitude in lead II, aVR, aVL, aVF, V3 and V5. Adjusted variable: sex, age, height, and weight.

**Table 4:** Comparison of general data between POTS to metoprolol response group and the non-response group ($\bar{x}$ ± s).

Variable	Non-Response Group	Response Group	*t*/*χ*^2^	*p* Value
Cases (n)	19	27	-	-
Sex (male/female)	13/6	20/7	0.18	0.675
Age (year)	12.31 ± 1.57	11.42 ± 2.67	−1.30	0.202
Height (cm)	160.39 ± 12.15	151.30 ± 18.44	−1.88	0.067
Weight (kg)	44.29 ± 9.46	40.24 ± 15.67	−1.00	0.321

**Table 5:** The predictive value of ventricular repolarization parameters in supine and standing ECGs for the prognosis of POTS to metoprolol [*M (P25, P75)*].

Variable	AUC (95% CI)	*p* Value	Sensitivity (%)	Specificity (%)	Cutoff Value	Youden Index
ΔT-wave amplitude in lead III (mV)	0.70 (0.54~0.86)	0.020	73.70	70.40	0.24	0.44
ΔT-wave amplitude in lead aVF (mV)	0.67 (0.40~0.91)	0.048	94.70	37.00	0.11	0.32
ΔT-wave amplitude in lead V2 (mV)	0.79 (0.65~0.93)	0.001	68.40	88.90	0.19	0.57
ΔT-wave amplitude in lead V3 (mV)	0.75 (0.60~0.91)	0.004	78.90	81.50	0.16	0.60
ΔT-wave amplitude in lead V4 (mV)	0.72 (0.56~0.87)	0.013	73.70	66.70	0.20	0.40
ΔT-wave amplitude in lead V5 (mV)	0.74 (0.59~0.89)	0.006	78.90	63.00	0.24	0.42
ΔTp-Te interval in lead V3 (ms)	0.74 (0.64~0.91)	0.002	52.60	96.30	29.50	0.49
ΔTp-Te/QT ratio in lead V3	0.82 (0.69~0.95)	0.000	63.20	92.60	0.05	0.56
Five combined indicators	0.93 (0.86~1.00)	0.035	94.70	81.50	-	0.76

The five combined indicators are ΔT-wave amplitude in lead leads V2, V3, and V5, ΔTp-Te interval in lead V3, and ΔTp-Te/QT ratio in lead V3 in supine and standing ECGs. AUC: area under the curve; CI: confidence interval.

## Data Availability

The original contributions presented in this study are included in the article; further inquiries can be directed to the corresponding author.

## Abbreviations

BP: blood pressure, BHUT: basic head-up tilt test, ECG: electrocardiogram, HR: heart rate, HUTT: head-up tilt test, POTS: postural orthostatic tachycardia syndrome, ΔHR: HR difference, ΔT-wave amplitude: T-wave amplitude differences, ΔTp-Te interval: Tp-Te interval difference, ΔTp-Te/QT ratio: Tp-Te/QT ratio difference.
